# Sensitization with vaccinia virus encoding H5N1 hemagglutinin restores immune potential against H5N1 influenza virus

**DOI:** 10.1038/srep37915

**Published:** 2016-11-28

**Authors:** Fumihiko Yasui, Yasushi Itoh, Ai Ikejiri, Masahiro Kitabatake, Nobuo Sakaguchi, Keisuke Munekata, Shintaro Shichinohe, Yukiko Hayashi, Hirohito Ishigaki, Misako Nakayama, Yoshihiro Sakoda, Hiroshi Kida, Kazumasa Ogasawara, Michinori Kohara

**Affiliations:** 1 Department of Microbiology and Cell Biology, Tokyo Metropolitan Institute of Medical Science, 2-1-6, Kamikitazawa, Setagaya-ku, Tokyo 156-8506, Japan; 2 Division of Pathology and Disease Regulation, Department of Pathology, Shiga University of Medical Science, Setatsukinowa, Otsu, Shiga 520-2192, Japan; 3 Department of Immunology, Nara Medical University, 840 Shijyomachi, Kashihara, Nara 634-8521, Japan; 4 WPI Immunology Frontier Research Center, Osaka University, 1-3 Yamadaoka, Suita, Osaka 565-0871, Japan; 5 Laboratory of Microbiology, Department of Disease Control, Graduate School of Veterinary Medicine, Hokkaido University, Sapporo, Hokkaido 060-0818, Japan

## Abstract

H5N1 highly pathogenic avian influenza (H5N1 HPAI) virus causes elevated mortality compared with seasonal influenza viruses like H1N1 pandemic influenza (H1N1 pdm) virus. We identified a mechanism associated with the severe symptoms seen with H5N1 HPAI virus infection. H5N1 HPAI virus infection induced a decrease of dendritic cell number in the splenic extrafollicular T-cell zone and impaired formation of the outer layers of B-cell follicles, resulting in insufficient levels of antibody production after infection. However, in animals vaccinated with a live recombinant vaccinia virus expressing the H5 hemagglutinin, infection with H5N1 HPAI virus induced parafollicular dendritic cell accumulation and efficient antibody production. These results indicate that a recombinant vaccinia encoding H5 hemagglutinin gene does not impair dendritic cell recruitment and can be a useful vaccine candidate.

H5N1 highly pathogenic avian influenza (HPAI) viruses are spreading among poultry at increasing rates worldwide. HPAI viruses represent a potential influenza pandemic, as would occur when a new virus emerges globally and infects individuals who have no immunity specific for new subtypes^1^. Human patients infected with H5N1 HPAI virus develop progressive pneumonia accompanied by diffuse alveolar damage and acute respiratory distress syndrome, as do macaques^2^,^3^. High levels of inflammatory cytokines were observed in the sera of human cases with severe H5N1 HPAI, suggesting that hypercytokinemia is involved in the pathogenicity of H5N1 HPAI virus in humans.

Favorable clinical outcome following influenza virus infection strongly depends on efficient production of neutralizing antibodies in virus-infected individuals. Thus, we hypothesized that the severity of H5N1 HPAI virus infection might reflect attenuation of immune responses necessary for efficient antibody production. Experimentally, antigen-captured macrophages appear in the marginal zone of germinal centers (GCs). In the marginal zone, IgD^+^ B-cells are stimulated with antigens to undergo T-cell-independent proliferation at extra-GCs and differentiation into antibody-producing cells^4^,^5^ or to become antigen-reactive B-cells that undergo proliferation in GCs^6^,^7^. Generally, influenza virus infection induces adaptive immune responses in peripheral lymphoid organs via T-cell-dependent processes. Antigens are captured by dendritic cells that migrate toward the T-cell zone of the white pulp region, where antigen-capturing dendritic cells activate T-cells. These activated T-cells in turn stimulate rapid proliferation of antigen-driven B-cells, thereby creating GCs in the follicular region^8^. The activated B-cells undergo affinity maturation and class switching toward IgG isotypes in GCs, and the B-cells producing high-affinity antibodies are selected and induced to differentiate into antibody-producing plasma cells or memory B-cells^9^,^10^,^11^,^12^. Therefore, antibody responses may be impaired if any of these processes are affected by H5N1 HPAI virus infection.

In the present study, we propose a mechanism that may account for impaired humoral immune responses against H5N1 HPAI viruses in comparison with those against H1N1 pdm viruses. As we report here, we observed a rapid loss of dendritic cells around B-cell follicles and in the T-cell zone following infection with H5N1 HPAI virus; this effect might reflect a fundamental defect in eliciting efficient adaptive immune responses with high-affinity neutralizing antibodies against H5N1 HPAI virus. This mechanism may explain why both mice and cynomolgus macaques infected with H5N1 HPAI virus showed more severe symptoms than did animals infected with the same viral titer of H1N1 pdm virus. We also showed, using mouse and non-human primate animal models, that a single immunization with a highly attenuated strain of vaccinia virus carrying an H5 hemagglutinin (HA)-encoding gene (rVV-H5 HA) was sufficient to prevent severe histological outcomes in animal lungs. In animals vaccinated with rVV-H5 HA, dendritic cells were recruited around B-cell follicles and T-cell zones. Taken together, our results suggest that H5N1 HPAI virus causes severe infection due to insufficient acquired immunity, reflecting impaired stimulation of T-cells by dendritic cells and insufficient induction of antigen-specific antibodies.

## Results

### Mice infected with H5N1 HPAI virus exhibit severe pneumonia with insufficient induction of humoral immune responses

**Figure 1:**
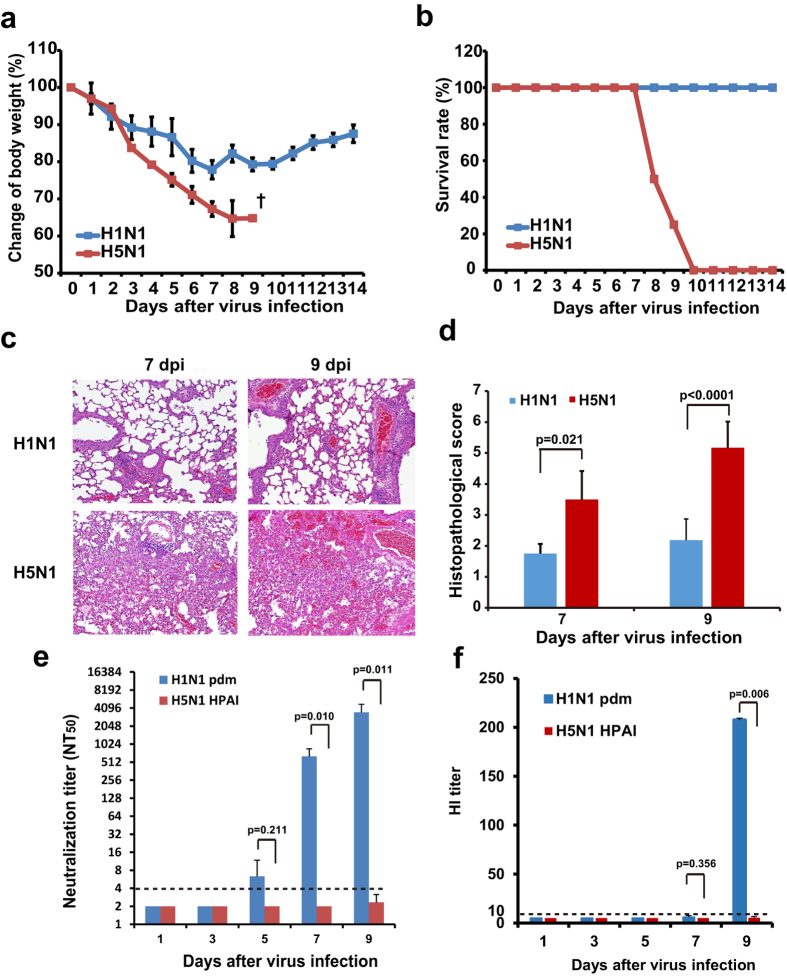
BALB/c mice infected with H5N1 HPAI virus show severe symptoms with insufficient antibody responses. BALB/c mice were infected intranasally with 1 × 10^4^ PFU of either H5N1 HPAI virus or H1N1 pdm virus. (**a**) Body weight was monitored daily in surviving animals following viral infection. (**b**) Survival rate was observed until 14 dpi. (**c**) Representative lung sections (hematoxylin and eosin staining; section thickness 4 μm) from H1N1 pdm virus-infected and from H5N1 HPAI virus-infected mice at 7 dpi and 9 dpi. For all micrographs, original magnification was 200×. (**d**) Histopathological scores were obtained at 7 dpi and 9 dpi from mice infected with H5N1 HPAI virus or H1N1 pdm virus. (**e**,**f**) Temporal changes in neutralization titer (**e**) and HI titer (**f**). Where applicable, data are presented as mean ± SD (n = 4–5). Broken lines indicate the detection limit of the assay. *p* values were calculated via Student’s t-test or Welch’s t-test.

Our experiments were designed to compare pathogenic processes that occur during H1N1 pdm and H5N1 HPAI viral infection in mice. Naïve BALB/c mice were infected with either H1N1 pdm virus or H5N1 HPAI virus at the same infectious dose (1 × 10^4^ PFU in a volume of 50 μL per mouse) (Fig. 1). The H5N1-infected mice exhibited marked decreases in body weight through day 9 (Fig. 1a), and all of these animals died or were humanely euthanized since symptoms reached a humane endpoint (see Methods) by 10 days post-infection (dpi) (Fig. 1b). In contrast, mice infected with H1N1 pdm virus exhibited moderate decreases in body weight before recovering without mortality (Fig. 1a,b). Pathological analyses demonstrated that the mice infected with H5N1 HPAI virus developed severe pneumonia with diffuse alveolar damage at 7 dpi and that the symptoms were markedly aggravated at 9 dpi (Fig. 1c). In contrast, the mice infected with H1N1 pdm virus showed only partial inflammation (Fig. 1c). The day-9 histopathological scores in the mice infected with H5N1 HPAI virus were significantly higher than those in the mice infected with H1N1 pdm virus (5.2 ± 0.8 vs. 2.2 ± 0.7 (mean ± SD), respectively; Fig. 1d).

To investigate differences in immune responses against H5N1 HPAI virus and H1N1 pdm virus infection in mice, we measured half-maximal titer of neutralizing antibody (NT_50_) and titer of hemagglutination inhibition (HI) against each virus. In mice infected with H1N1 pdm virus, the NT_50_ titer increased markedly from 5 dpi, reaching a mean value of 3487 ± 1250 at 9 dpi (Fig. 1e). The HI titer achieved a mean value of 207.5 ± 1.7 at 9 dpi (Fig. 1f). In contrast, the NT_50_ and HI titers in all H5N1 HPAI-infected mice (with the exception of one H5N1-infected mouse) remained below the limits of detection (4 in NT_50_ titer and 10 in HI titer; Fig. 1e and f) during the experimental period.

### Cynomolgus macaques infected with H5N1 HPAI virus exhibit severe pneumonia with insufficient induction of humoral immune responses

**Figure 2:**
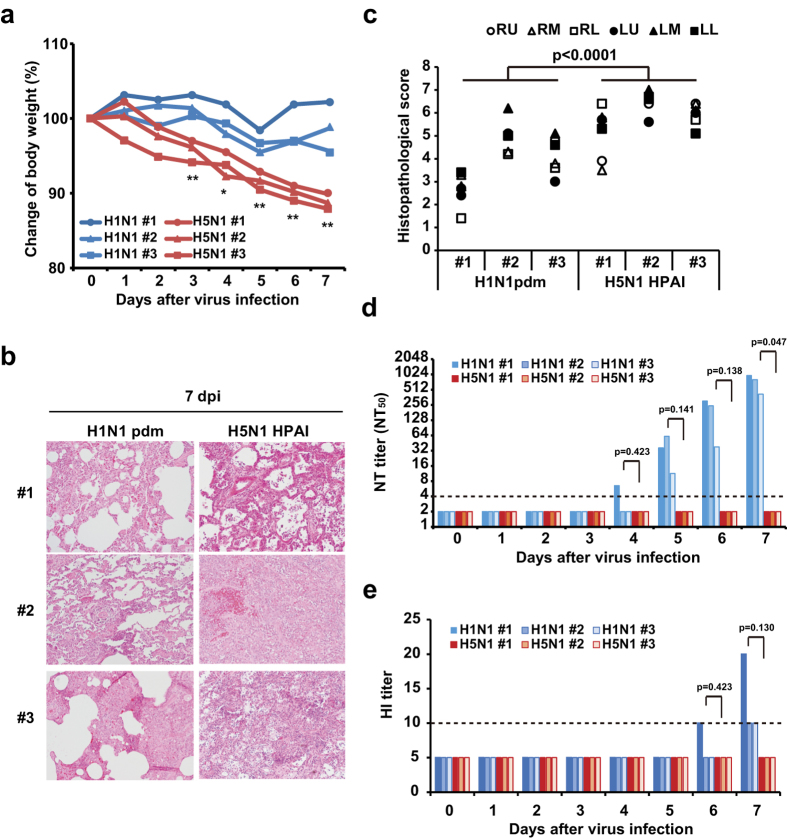
Cynomolgus macaques infected with H5N1 HPAI virus show severe symptoms with insufficient antibody responses. Female cynomolgus macaques were inoculated intradermally with a LC16m8-derived recombinant vaccinia virus that does not encode an influenza HA protein (rVV-Empty) and then were infected 4 weeks later with 3 × 10^6^ PFU of either H5N1 A/whooper swan/Hokkaido/1/2008 strain (H5N1 HPAI virus, n = 3) or H1N1 A/Narita/1/2009 strain (H1N1 pdm virus, n = 3) by intratracheal, intranasal, and tonsillar routes. (**a**) Body weight was monitored daily after virus infection. # values indicate individual animal numbers. *p* values were calculated via Student’s t-test. *p < 0.05, **p < 0.01. (**b**) Representative lung sections (hematoxylin and eosin staining; section thickness 4 μm) from H1N1 pdm virus-infected and from H5N1 HPAI virus-infected macaques at 7 dpi. For all micrographs, original magnification was 200×. (**c**) Histopathological scores were obtained for each of several defined regions from each animal’s lungs (right upper, RU; right middle, RM; right lower, RL; left upper, LU; left middle, LM; and left lower, LL) at 7 dpi with H5N1 HPAI virus or H1N1 pdm virus. *p* values were calculated via Student’s t-test. (**d**,**e**) Temporal changes of neutralization titer (**d**) and HI titer (**e**) were measured. Broken lines indicate the detection limit in the assay. *p* values were calculated via Welch’s t-test.

To determine whether the differences in responses depended on the species of the host animal, we investigated immune responses in the pathogenesis of H1N1 pdm and H5N1 HPAI viruses in cynomolgus macaques. Control macaques were inoculated with recombinant vaccinia virus (rVV)-Empty, which did not include any influenza virus gene, and were then infected with H5N1 HPAI virus or H1N1 pdm virus. The body weights of three macaques infected with H5N1 HPAI virus fell significantly compared with those of three macaques infected with H1N1 pdm virus (Fig. 2a). Pathologically, the lungs of the macaques infected with H5N1 HPAI virus exhibited diffuse alveolar damage, including interstitial inflammation and alveolar edema. In contrast, the macaques infected with H1N1 pdm virus exhibited only localized pulmonary inflammation, even though each macaque was infected with the same titer of the respective virus (Fig. 2b). At 7 dpi, the mean histopathological score of six lung lobes in individual macaques infected with the H5N1 HPAI virus was significantly higher than that in macaques infected with H1N1 pdm virus (Fig. 2c; *P* < 0.0001, H1N1 vs. H5N1). We next measured the NT_50_ and HI titers against each virus. In the macaques infected with H1N1 pdm virus, the NT_50_ titer exhibited a nominal increase from 5 dpi, reaching 418–974 at 7 dpi (Fig. 2d). The HI titer also increased from 6 dpi, and HI activity was observed in all (3/3) H1N1 pdm virus-infected macaques at 7 dpi (Fig. 2e). In contrast, neither NT_50_ titer nor HI titer was detected in any (0/3) macaques infected with H5N1 HPAI virus during the experimental period (Fig. 2d and e). These results suggest that the antibody responses against H5N1 HPAI virus infection were severely reduced in both mice and macaques.

### H5N1 HPAI virus infection impairs mature GC formation

**Figure 3:**
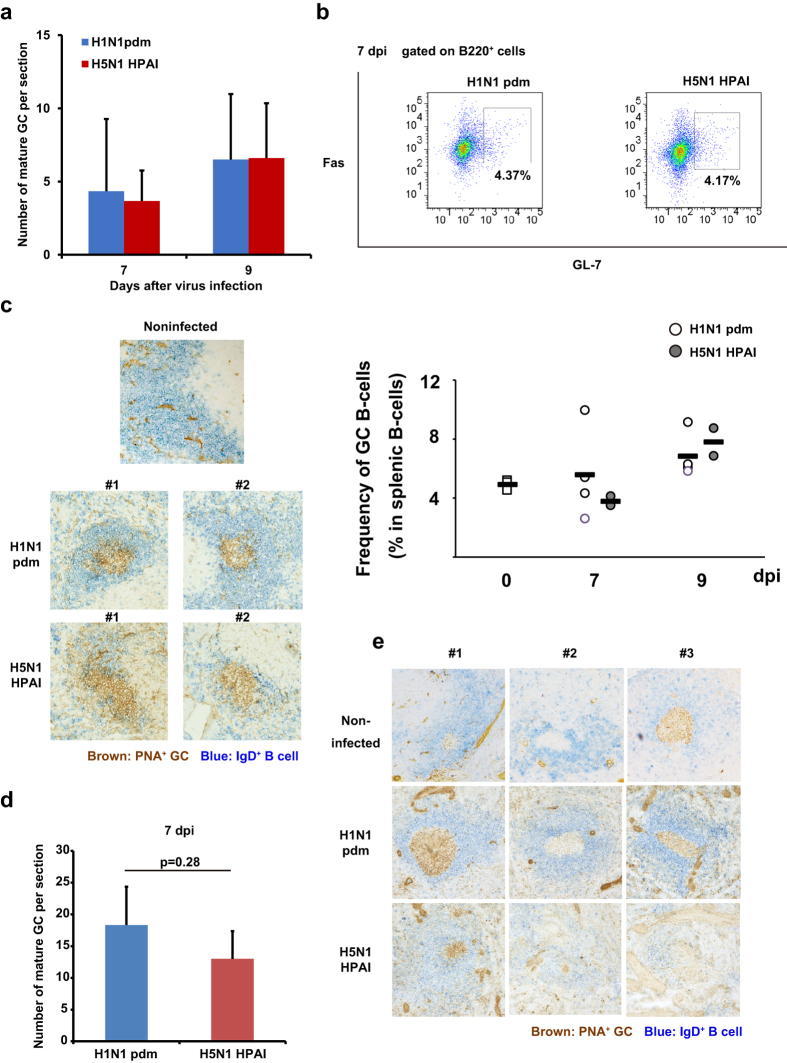
H5N1 HPAI virus-infected mice and macaques exhibit changes in formation of splenic B-cell follicles. (**a**) The number of germinal centers (GCs) formed in each section of mouse spleen was measured at 7 dpi and 9 dpi. (**b**) The percentage of B220^+^, GL-7^+^, Fas^+^ GC B-cells in the spleens infected with either H5N1 HPAI virus or H1N1 pdm virus was analyzed by flow cytometry at 0, 7, and 9 dpi (n = 4, except n = 2 at 9 dpi in the H5N1 HPAI virus-infected group). Control samples were obtained from non-infected BALB/c mice. (**c**) The formation of GCs (stained for PNA; brown) and B-cell follicles (IgD^+^; blue) in mice infected with either H5N1 HPAI virus or H1N1 pdm virus was analyzed by immunohistochemical staining at 9 dpi. Representative images of B-cell regions are provided; original magnification was 400×. (**d**) The number of GCs formed in each slice of macaque spleen was measured at 7 dpi. Where applicable, data are presented as mean ± SD (n = 3). (**e**) The formation of GCs and B-cell follicles in the macaque spleens was analyzed by immunohistochemical staining at 7 dpi with either H5N1 HPAI virus or H1N1 pdm virus. Control samples were obtained from non-infected macaques. Representative images of B-cell regions are provided; original magnification was 400×. *p* values were calculated via Student’s t-test. **p < 0.01.

To clarify the mechanism underlying the impaired induction of humoral immune responses in the mice and macaques infected with H5N1 HPAI virus, we investigated the formation of B-cell follicles in the spleen after infection. The mean number of heterogeneous peanut agglutinin (PNA)-binding GCs (PNA^+^ GCs) in each section in mice infected with H5N1 HPAI virus was similar to that in mice infected with H1N1 pdm virus (Fig. 3a; Supplementary Fig. 1). The percentages of GC B-cells (B220^+^, GL-7^+^, Fas^+^) in the spleens were also similar between the mice infected with H5N1 HPAI virus and those infected with H1N1 pdm virus when assessed at 7 and 9 dpi (Fig. 3b). However, B-cell follicles in mice infected with H5N1 HPAI virus showed a loose and thin outer layer of IgD^+^ cells (Fig. 3c, lower panels). In marked contrast, B-cell follicles in mice infected with H1N1 pdm virus were visualized as clear PNA^+^ GCs surrounded by a dense layer of IgD^+^ B-cells (Fig. 3c, upper panels). At 7 dpi, the mean number of GCs in each section in the H1N1 pdm virus-infected macaques was nominally higher than that in the H5N1 HPAI virus-infected macaques, although the difference was not statistically significant (Fig. 3d; Supplementary Fig. 2). As seen in mice, a loose and thin outer layer of B-cell follicles was observed in the spleens of macaques infected with H5N1 HPAI virus, but not in the spleens of H1N1 pdm-infected macaques (Fig. 3e). As the uninfected macaques used in this study were grown under conventional conditions, slight GC formation was observed even in the spleens of uninfected macaques (Fig. 3e). The staining intensity of splenic IgD^+^ B-cells in macaques infected with H5N1 HPAI virus was markedly lower than that of splenic IgD^+^ B cells in uninfected control macaques. Taken together, these results implied that infection with H5N1 HPAI virus decreases the maturation of follicular B-cells in the spleen, and suggested that antigen-specific B-cell responses to H5N1 HPAI virus infection may be impaired in mice and cynomolgus macaques.

### Decrease of CD11c^+^ dendritic cells in the spleen T-/B-cell border during immune responses to H5N1 HPAI virus infection in mice

**Figure 4:**
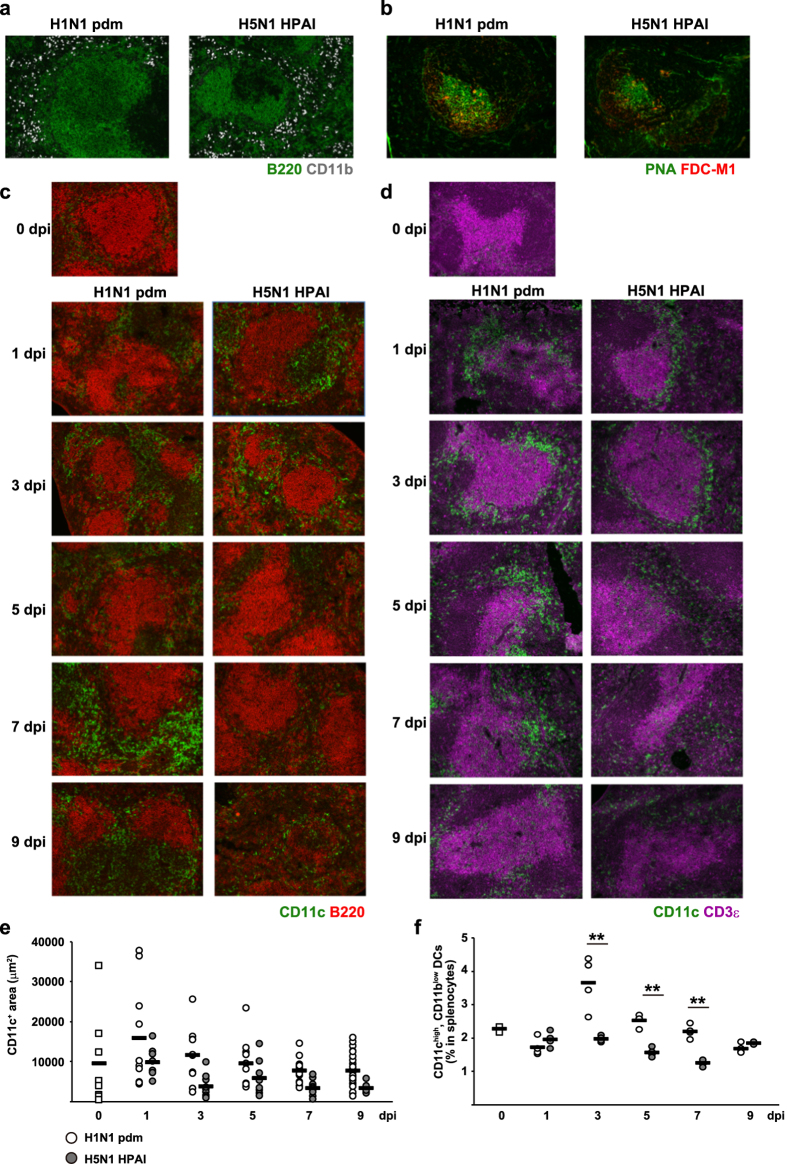
Accumulation of dendritic cells surrounding B-cell follicles and T-cell zone is weaker in H5N1 HPAI virus-infected mice than in H1N1 pdm virus-infected mice. (**a**) Immunofluorescence staining of the spleen was performed at 7 dpi for mice infected with either H1N1 pdm virus or H5N1 HPAI virus. Macrophages (CD11b^+^; white) were observed in the marginal zone around B-cell follicles (B220^+^; green). (**b**) Follicular dendritic cells (FDC-M1; red) accumulated in the germinal center (PNA; green). (**c**) Localization of dendritic cells (CD11c^+^; green) and B-cells (B220^+^; red) in the spleen of mice infected with either H5N1 HPAI virus or H1N1 pdm virus was analyzed at 0, 1, 3, 5, 7, and 9 dpi by immunofluorescent staining. (**d**) Localization of dendritic cells (CD11c^+^; green) and T-cells (CD3ε^+^; magenta) in the spleen of mice infected with either H5N1 HPAI virus or H1N1 pdm virus was analyzed at 0, 1, 3, 5, 7, and 9 dpi by immunofluorescent staining. (**e**) The area of dendritic cells in and around the T-cell zone was calculated by BZ-X Analyzer at a magnification of 200× (n = 3–5 mice per group). Horizontal bars indicate mean values (n = 8–24). Individual micrographs are representative data from the indicated time point. (**f**) The percentage of CD11c^high^, CD11b^low^ dendritic cells of splenocytes was analyzed at 0, 1, 3, 5, 7, and 9 dpi by flow cytometry (n = 4 mice per group, except n = 2 at 9 dpi in H5N1 HPAI virus-infected mice). **p < 0.01.

To investigate the initial triggering of antibody responses against H5N1 HPAI virus infection, we analyzed localization of antigen-presenting cells (APCs), including dendritic cells and macrophages, in the spleen. Unfortunately, in our hands, available antibodies did not permit recognition of dendritic cell-specific markers in immunofluorescent staining of frozen macaque spleen sections (data not shown). Nonetheless, BALB/c mice infected with H5N1 HPAI virus exhibited symptoms similar to those observed in macaques infected with H5N1 HPAI virus (Fig. 3c and e). Therefore, we compared the localization and percentage of APCs in the spleens of BALB/c mice infected with H5N1 HPAI virus to those of mice infected with H1N1 pdm virus. Throughout the infection period, localization of CD11b^+^ macrophages (CD11b^+^; white) in the marginal zone of spleens of mice infected with H5N1 HPAI virus was similar to that in mice infected with H1N1 pdm virus (Fig. 4a). The percentage of macrophages (CD11c^low^, CD11b^high^, F4/80^+^) in the spleen in the H5N1 HPAI virus-infected group was similar to that in the H1N1 pdm virus-infected group (Supplementary Fig. 3). No significant change in the localization of follicular dendritic cells (FDC-M1^+^; red) in GCs (PNA^+^; green) or in the percentage of this cell population (FDC-M2^+^, ICAM-1^+^) was seen following infection with either of these viruses (Fig. 4b; Supplementary Fig. 4). In H1N1 pdm virus-infected animals, CD11c^+^ dendritic cells (CD11c^+^; green) accumulated around the B-cell (B220^+^; red) follicles from 0 dpi through 9 dpi (Fig. 4c; Supplementary Fig. 5). At 1 dpi and 3 dpi, the pattern of accumulation of CD11c^+^ dendritic cells following H1N1 pdm virus infection appeared similar to that following H5N1 HPAI virus infection. However, accumulation of CD11c^+^ dendritic cells around the B-cell follicles in the spleens of H5N1 HPAI virus-infected mice appeared to decrease after 5 dpi. In mice infected with the H1N1 pdm virus, dense staining for CD11c^+^ dendritic cells was observed in the parafollicular area of CD3ε T-cells (CD3ε^+^; magenta) throughout the infection period (Fig. 4d, left), with CD11c^+^ dendritic cells being particularly abundant until 7 dpi. CD11c^+^ dendritic cells surrounded the T-cell clusters in the parafollicular area at the initial period of H5N1 HPAI virus infection, but the layer of CD11c^+^ dendritic cells appeared thinner and lower in number than that observed with H1N1 pdm virus infection. Notably, the area and number of dendritic cells surrounding the parafollicular area of the T-cells at 5 dpi were smaller in mice infected with H5N1 HPAI virus than those in mice infected with H1N1 pdm virus (Fig. 4d and e; Supplementary Fig. 6). The percentage of dendritic cells (CD11c^high^, CD11b^low^) in the spleens of mice infected with H5N1 HPAI virus also was statistically lower than that in the spleens of mice infected with H1N1 pdm virus (Fig. 4f). In addition, a significant decrease in the number of dendritic cells (CD11c^high^, F4/80^−^) was observed in the lung (the site of infection) in mice infected with H5N1 HPAI virus compared to the number in the lung of mice infected with H1N1 pdm virus (Supplementary Fig. 7).

### Looser and rougher cell-cell interaction of dendritic cells with T-cells in the spleens of mice infected with H5N1 HPAI virus compared to that in mice infected with H1N1 pdm virus

**Figure 5:**
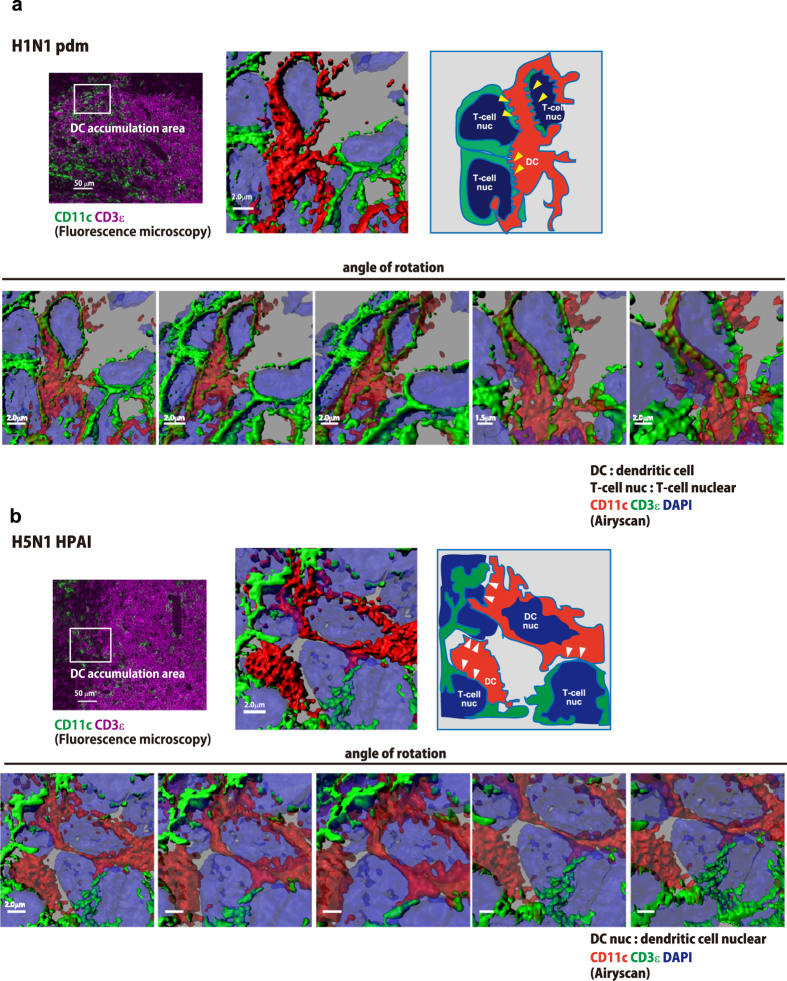
High-resolution microscopy reveals fewer and looser cell-cell interactions between dendritic cells and T-cells in the spleens of mice infected with H5N1 HPAI virus. High-resolution micrographs were obtained from sections of spleen recovered (at 7 dpi) from mice infected with H1N1 pdm virus (**a**) or H5N1 HPAI virus (**b**). Images were visualized by Imaris software and are representative data. Sections were stained for CD11c (red), CD3ε (green), and nuclear DNA (DAPI; blue). To clarify the interaction of dendritic cells with T-cells, the angle of rotation was changed (lower panels). Yellow arrowheads indicate tight and intricate interactions (**a**); white arrowheads indicate loose and rough interactions (**b**). To enhance image clarity, the transparency of the CD11c rendering was set to 60% (lower panels) and the transparency of DAPI was set to 78% (all panels).

Since a decrease in dendritic cell accumulation was observed in the spleens of mice infected with H5N1 HPAI virus (Fig. 4d,e and f), we focused on the cell-cell interaction of dendritic cells with T-cells. The accumulation area of dendritic cells in fluorescence photomicrographs (Fig. 5a and b) was analyzed by high-resolution microscopy using an Airyscan-enabled LSM800 and 880 confocal microscopes. The acquired z-stack data were reconstituted by Imaris software and visualized as a three-dimensional image. In the spleen of naïve mouse, dendritic cells were located adjacent to T-cells (Supplementary Fig. 8). However, at 7 dpi, splenic CD11c^+^ dendritic cells in mice infected with H1N1 pdm virus displayed tight and intricate (jigsaw puzzle-like) contacts with T-cells (Fig. 5a, yellow arrowheads; Supplementary movie 1). In contrast, the cell-cell interactions of dendritic cells with T-cells were fewer and rougher in the spleens of mice infected with H5N1 HPAI virus (Fig. 5b, white arrowheads; Supplementary movie 2). Previous studies showed that the tight interaction of dendritic cells with T-cells plays an important role in the activation of antigen-specific B-cells to produce high-affinity anti-viral antibodies^13^,^14^. Therefore, our results suggest that animals infected with H5N1 HPAI virus were not able to induce high-affinity antibodies against target viruses because of insufficient cell-cell interactions among antigen-presenting cells (such as dendritic cells with T-cells), thus failing to provide sufficient stimuli to naïve B-cells.

### Pre-sensitization with recombinant vaccinia virus expressing H5 HA protein potentiates efficient humoral immune responses after H5N1 HPAI virus challenge by inducing the accumulation of dendritic cells

**Figure 6:**
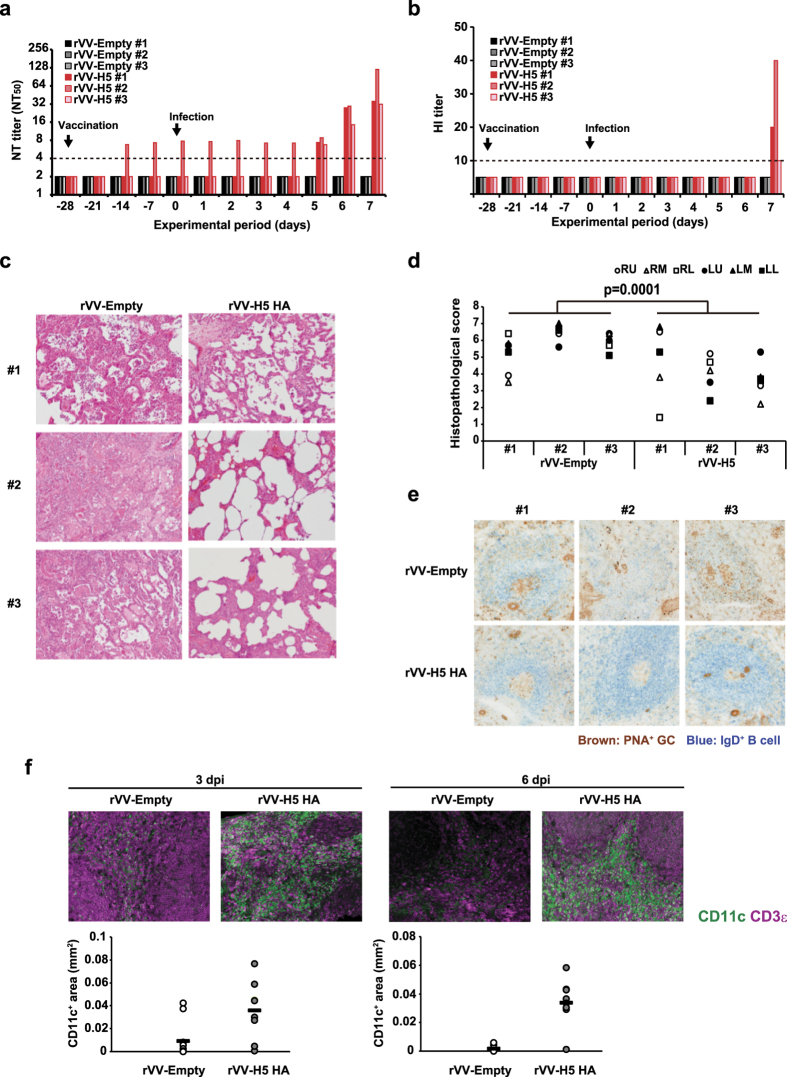
Pre-sensitization of macaques with vaccinia virus-encoded recombinant H5 HA induces sufficient humoral immune responses after H5N1 HPAI virus challenge. Macaques were inoculated intradermally with 1 × 10^7^ PFU per animal of rVV-Empty or rVV-H5 HA (i.e., lacking insert or encoding H5 HA, respectively). rVV-Empty-inoculated macaques were the same individuals that were assessed as H5N1 HPAI virus-infected macaques in Fig. 2. (**a**,**b**) Time courses of the titers of neutralizing antibody (**a**) and HI (**b**) were measured. Broken lines indicate the detection limit of the assay. (**c**) Representative lung sections (hematoxylin and eosin staining; section thickness 4 μm) at 7 dpi; original magnification was 200×. (**d**) Histopathological scores were obtained for each of several defined regions from each animal’s lungs (RU, right upper; RM, right middle; RL, right lower; LU, left upper; LM, left middle; LL, left lower) at 7 dpi with rVV-Empty or rVV-H5HA. *p* values were calculated via Student’s t-test. (**e**) Representative images of B-cell region (GCs, brown; IgD, blue; section thickness 6 μm) in the spleen of macaques inoculated with rVV-Empty and rVV-H5 HA at 7 dpi; original magnification was 400×. (**f**) Upper panel: Localization by immunofluorescent staining of dendritic cells (CD11c; green) and T-cells (CD3ε; magenta) in the spleen of mice immunized with either rVV-H5 HA or rVV-Empty and then infected with H5N1 HPAI virus. Spleens were recovered at 3 or 6 dpi. Lower panel: The area of dendritic cells in the T-cell zone of these spleens was calculated using the BZ-X Analyzer at a magnification of 200× (n = 3).

To reinforce antibody responses of macaques infected with H5N1 HPAI virus, macaques were immunized intradermally with a single dose of rVV-H5 HA, a recombinant vaccinia virus (derived from the highly attenuated strain LC16m8)^15^ that harbors the gene encoding the H5 hemagglutinin (HA) protein (Clade 2.3.4) under control of an ATI/p7.5 synthetic hybrid promoter^16^,^17^,^18^,^19^. Following subsequent H5N1 HPAI virus infection, macaques primed with rVV-H5 HA displayed formation of mature GCs (with an apparent outer layer of IgD^+^ B-cell follicles) in a manner that resembled that seen following H1N1 pdm virus infection (Fig. 6e). Inoculation with rVV-H5 HA also induced production of a neutralizing antibody titer (NT_50_ = 6.7) in one of three macaques when assessed at 2 weeks after immunization. This rVV-H5 HA-induced titer was sustained through the subsequent challenge with H5N1 HPAI virus (Fig. 6a). Notably, all (3/3) macaques pre-sensitized with rVV-H5 HA showed rapid increases in titers of neutralizing antibodies from 5 dpi to 7 dpi following challenge with H5N1 HPAI virus. This effect was observed even in the macaques in which neutralizing antibodies were not detected before the virus challenge (Fig. 6a). In contrast, in control macaques immunized with rVV-Empty, no neutralizing antibody was detected following challenge with H5N1 (Fig. 6a; data were obtained from the experiments in Fig. 2d). The effect of pre-sensitization was confirmed in an assay of the HI titers in macaques following H5N1 virus challenge (Fig. 6b; data for rVV-Empty were obtained from the experiments in Fig. 2e). At 7 dpi with H5N1 HPAI virus, lung pathology of pre-sensitized macaques was ameliorated significantly compared with that of the control macaques immunized with rVV-Empty (Fig. 6c and d; data for rVV-Empty were obtained from the experiments in Fig. 2c). The macaques inoculated with rVV-H5 HA showed recovery of mature GC formation with an apparent outer layer of IgD^+^ B-cell follicles after H5N1 HPAI infection (Fig. 6e). Furthermore, accumulation of dendritic cells surrounding the parafollicular area of T-cells during H5N1 HPAI virus infection of mice was restored by prior immunization with rVV-H5 HA (Fig. 6f). These results suggested that pre-sensitization with H5 HA antigen reduces the severity of symptoms caused by infection with H5N1 HPAI virus, presumably by restoration of tight interactions of dendritic cells with T-cells and induction of antigen-specific neutralizing antibodies.

## Discussion

The clinical mortality of H5N1 HPAI virus infection exceeded 50% among individuals infected between 2003 and 2014 in more than twelve countries^20^. The severity of systemic symptoms appears to result from a “cytokine storm”, in which high levels of inflammatory cytokines are secreted in patient blood. Previous studies using a macaque model demonstrated that innate immune responses are dysregulated during infection by H5N1 HPAI virus compared to those obtained during infection by low-pathogenicity influenza virus^21^,^22^. In addition, Hatta *et al*. reported that the CD8^+^ T-cell response was ineffective in controlling viral replication in mice infected with H5N1 HPAI virus^23^. However, the implication of adaptive immune responses in the severity of H5N1 HPAI virus infection is not completely understood.

In the present study, we observed an abnormality in antibody responses in both mice and cynomolgus macaques following infection with H5N1 HPAI virus. Namely, H5N1 HPAI virus-infected mice and macaques exhibited a decreased antibody response to H5N1 HA, reflecting a lack of cell-cell interaction between dendritic cells and T-cells, and consistent with impaired formation of outer layers of B-cell follicles in the secondary lymphoid organs of these animals. Usually, innate and acquired immunity cooperate to eliminate influenza virus from the host. The present study emphasizes the significant role of acquired immunity against H5N1 HPAI virus. Notably, mice infected with H5N1 HPAI virus died (or had to be humanely sacrificed) at around 10 dpi, a time point at which acquired immunity is more important than innate immunity. Previous reports by Katz *et al*.^24^,^25^ and by Kitajima *et al*.^26^ employed dose-response-time modeling (in BALB/c mice and ferrets) to analyze changes in immune responses and mortality rates after H5N1 influenza virus infection. These papers optimized the experimental data set to obtain representative mortality rates at 7 to 10 dpi with 2–3 viral dose levels [log_10_ EID_50_]. In contrast, H1N1 viruses (e.g., PR8) cause mortality in mice within 5 dpi, suggesting the importance of innate immunity in the immunological response to PR8^27^. A difference in immune response against H5N1 HPAI virus (compared to that against H1N1 virus) also has been reported in a macaque model^21^,^28^; that study focused on dysregulation of innate immune responses. Thus, the contribution to pathogenicity of acquired immunity against H5N1 HPAI virus has not been well examined. Notably, high-affinity and neutralizing anti-influenza virus antibodies are generated after B-cell maturation. Our investigation, which compared murine immune responses against H5N1 to those against H1N1, was performed using carefully selected viral titers. Additionally, for our experimental data set, the responses against H1N1 and H5N1 were compared in parallel, that is, at the same number of dpi. We also carefully examined all mice throughout the infection interval before sacrificing the animals for intensive *ex vivo* pathological and immunological analyses.

Using a non-human primate model, we showed that macaques infected with H5N1 HPAI virus did not induce sufficient humoral immune responses, especially neutralizing antibodies, against H5N1 HPAI virus compared to the responses observed in macaques infected with H1N1 pdm virus. Insufficient induction of antibody response also was observed in BALB/c mice after H5N1 HPAI virus infection. Furthermore, decreased accumulation of dendritic cells at the interface between the marginal zone and parafollicular area was observed prior to impaired formation of B-cell follicles in H5N1-infected animals, raising the possibility that activation of B-cells specific for H5N1 HPAI virus failed, presumably because of insufficient cell-cell interaction of dendritic cells with T-cells.

In the present study, dendritic cells accumulated by 3 dpi in the spleens of mice infected with H5N1 HPAI virus or H1N1 pdm virus. However, subsequent differences in dendritic cell distribution were apparent by 7 dpi. The migration of dendritic cells to the spleen in an early post-infection phase was similar in both viral infections, but accumulation of dendritic cells may have been decreased at the interface between B-cell follicles and the parafollicular area in the H5N1-infected animals. Functional interaction between Th-cells and dendritic cells is suppressed by activation-induced cell death following infection with numerous viruses^29^,^30^,^31^,^32^,^33^. Following infection of macaques with H5N1 HPAI virus, dendritic cells in the blood of these animals underwent apoptosis in the draining lymph nodes^34^. Additionally, cell death of human dendritic cells has been induced after *in vitro* infection with H5N1 HPAI virus^32^. Other work has shown that differentiation of follicular helper T-cells (TFH cells), which regulate high-affinity B-cell proliferation, was mediated by conventional dendritic cells^35^. Thus, disappearance of dendritic cells from the interface between the marginal zones and the parafollicular area, along with loose and rough cell-cell interaction of dendritic cells with T-cells (as observed in the present work), are predicted to result in reduction of antibody responses after H5N1 HPAI virus infection.

The molecular mechanism associated with insufficient residence of dendritic cells around the follicular region in the H5N1 HPAI virus-infected macaques and mice remains unknown. However, one possible mechanism is that infection of immune-competent dendritic cells (or other accessory cells) with H5N1 HPAI virus might directly deplete dendritic cells in the parafollicular region^32^. Alternatively, virus-infected cells may exhibit altered surface expression of FAS, CD40, CD80, CD86, and/or anti-apoptotic molecules such as intracellular Bcl-2^36^,^37^,^38^. Under those circumstances, apoptosis of dendritic cells might be induced by the virus-infected cells.

Immunization with rVV-H5 HA induced sufficient initial immune responses with tight cell-cell interactions between dendritic cells and T-cells, along with activation of follicular B-cells to form mature GCs. In this context, the rVV-H5 HA virus (which does not encode other components of the H5N1 HPAI virus) has several advantages as a potential H5N1 vaccine. Notably, the rVV-H5 HA virus rapidly provides immunogenicity with a single dose; additionally, rVV-H5 HA enhances the effective interaction of dendritic cells with T-cells following H5N1 HPAI virus infection. Time-course data for viral titer in mouse lungs demonstrated that rVV-H5 immunization could decrease the viral load of H5N1 HPAI virus in the infection sites in the early phase of H5N1 HPAI virus infection (Supplementary Fig. 9). Considering these results, we hypothesize that immunization with rVV-H5 might alleviate the decrease in dendritic cell-interaction caused by H5N1 HPAI virus infection in mice, resulting in tight cell-cell interaction between dendritic cells and T-cells and subsequent formation of mature GCs. The efficacy of protective immunity mediated by recombinant vaccinia viruses encoding the H5N1 HA gene also has been reported using another vaccinia virus strain (MVA). Kreijtz *et al*. have reported that immunization with a MVA-based H5N1 vaccine can induce cross-reactive antibodies, including cross-clade protection (in mice and macaque monkeys) against heterologous H5N1 influenza virus^39^,^40^. In the present study, we not only confirmed impaired formation of B-cell follicles after H5N1 influenza virus infection, but also showed potentiation of the activation of dendritic cell/T-cell interaction (and improved antibody responses) in H5N1-infected animals previously immunized with rVV-H5N1-HA. Our previous studies and those of others have demonstrated the induction of memory T-cell and B-cell responses following vaccination with recombinant vaccinia virus^16^,^17^,^18^,^19^. Further studies will be needed to clarify the efficacy of T-cell-mediated responses in the alleviation of H5N1 HPAI virus infection-associated symptoms following vaccination with recombinant vaccinia virus. Our results demonstrate that immunization with rVV-H5 is sufficient to enhance protective activity against infection by the H5N1 virus. LC16m8-derived recombinant vaccinia viruses engineered (like rVV-H5 HA) to express genes encoding particular influenza HA proteins may serve as promising vaccine candidates.

## Methods

### Ethics statement

All experiments using primary rabbit kidney (pRK) cells and mice were approved by the Tokyo Metropolitan Institute of Medical Science Animal Experiment Committee (Permission Number: 22-47-2) and were performed in accordance with the animal experimentation guidelines of the Tokyo Metropolitan Institute of Medical Science. All macaque experiments were approved by the Shiga University of Medical Science Animal Experiment Committee and the Recombinant Gene Experiment Safety Committee (Permission Number: 2011-6-8 H), and were performed in accordance with the *Guidelines for the Husbandry and Management of Laboratory Animals* of the Research Center for Animal Life Science at Shiga University of Medical Science, and with *Fundamental Guidelines for Proper Conduct of Animal Experiment and Related Activities in Academic Research Institutions* under the jurisdiction of the Ministry of Education, Culture, Sports, Science and Technology, Japan.

### Viruses and cells

H1N1 pdm influenza virus A/Narita/1/2009 strain (kindly provided by Dr. Takato Odagiri, National Institute of Infectious Diseases, Japan), A/Tokyo/2619/2009 strain, and H5N1 HPAI virus A/whooper swan/Hokkaido/1/2008 strain were used in this study. H5N1 A/whooper swan/Hokkaido/1/2008 was grown in embryonated eggs. Virus-containing allantoic fluid was harvested and stored in aliquots at −80 °C until use. H1N1 A/Narita/1/2009 strain and A/Tokyo/2619/2009 strain were subcultured in MDCK cells grown in MEM (Nissui Pharmaceutical Co., Ltd., Tokyo, Japan) containing 1% bovine serum albumin (BSA) and 10 μg/mL acetyl-trypsin. All procedures using H5N1 HPAI virus were performed in biosafety level 3 facilities by personnel wearing powered air-purifying respirators (Shigematsu Co., Ltd., Tokyo, Japan).

Vaccinia virus strain LC16m8 was kindly provided by KAKETSUKEN Co., Ltd. (Kumamoto, Japan). In preparation for the construction of LC16m8-derived recombinant vaccinia virus (engineered to express the H5N1 influenza virus hemagglutinin (HA) protein), LC16m8 was propagated on primary rabbit kidney (pRK) cell cultures prepared as follows. Kidneys extirpated from 7-day-old inbred JW rabbits (KITAYAMA LABES Co., Ltd., Nagano, Japan) were fragmented and subjected to overnight digestion with 100 PU/mL dispase (Sanko Jun-yaku Co., Ltd., Tokyo, Japan). The cells were grown in 150-mm dishes in lactalbumin medium with Hanks’ salts (LH) supplemented with 10% newborn calf serum (NCS), 100 U/mL penicillin, and 100 μg/mL streptomycin. When the cell confluency was approximately 50%, the culture medium was replaced with lactalbumin medium with Eagle’s salts (LE) supplemented with 10% NCS, 100 U/mL penicillin, and 100 μg/mL streptomycin. These cultures were infected with LC16m8 as indicated in the next section.

### Generation of recombinant vaccinia virus

Synthetic DNA encoding multibasic-site-deleted hemagglutinin protein of H5N1 HPAI virus A/Anhui/01/2005(H5N1)-PR8-IBCDC-RG5 influenza virus [Clade 2.3.4] was purchased from Sloning BioTechnology (Puchheim, Germany). The synthetic DNAs were amplified by PCR using primers that incorporated flanking restriction enzyme sites (*Sbf* I and *Sgf* I). The amplicons were double-digested with *Sbf* I and *Sgf* I, and then ligated into a similarly double-digested pBMSF7c vector. The resulting construct (pBMSF7c-mCl2.3) harbored the HA-encoding ORF downstream of an ATI/p7.5 hybrid promoter^18^. pBMSF7c-mCl2.3 was linearized with restriction enzymes and transfected into pRK cells (prepared as above) that had been infected with vaccinia virus LC16m8 at a multiplicity of infection (moi) of 10 for 1 h. After 24 h, the virus-cell mixture was harvested by scraping, and frozen at −80 °C until use. Because the HA protein of influenza virus, but not that of vaccinia virus, can aggregate guinea pig erythrocytes, the resulting HA-positive recombinant viruses able to recognize guinea pig erythrocytes were purified 3 times by plaque cloning; the resulting viral stock was designated rVV-H5 HA. Expression of influenza virus HA protein from rVV-H5 HA vaccines was confirmed by western blotting. A control recombinant vaccinia virus, rVV-Empty, was generated by an analogous procedure using transfection and homologous recombination with linearized empty vector pBMSF7c (rather than pBMSF7c-mCl2.3). The resulting rVV-Empty contained the ATI/p7.5 hybrid promoter but not a transgene encoding an influenza HA protein. rVV-Empty was purified as a HA-negative recombinant vaccinia virus by plaque cloning using chicken erythrocytes.

### Mouse study

Mice had free access to food and water and were maintained on a 12-h light/12-h dark cycle. Prior to inoculation, animals were anesthetized by intraperitoneal administration of 0.15 mL/mouse of a ketamine-xylazine mixture. Nine-week-old female BALB/c mice (SLC, Shizuoka, Japan) were inoculated intranasally with 1 × 10^4^ PFU of H5N1 A/whooper swan/Hokkaido/1/2008 or H1N1 A/Tokyo/2619/2009 strain. Clinical signs were monitored for 14 days post-infection (dpi) and mice were sacrificed at 1, 3, 5, 7, and 9 dpi to collect organ samples and sera. Body weight was monitored daily; mice that lost 25% or more of their initial body weight were scored as dead and humanely euthanized. For the vaccination experiments, the mice were inoculated intradermally (in their backs, using a two-step needle) with either 1 × 10^7^ PFU (1 × 10^6^ PFU/5 μl × 10) of rVV-H5 HA or rVV-Empty. At 5 weeks after vaccination, the mice were challenged intranasally with 1 × 10^4^ PFU of H5N1 A/whooper swan/Hokkaido/1/2008 strain. Animals were sacrificed and then the spleens and the lungs were collected at 1 (for lungs), 3 or 6 dpi.

### Macaque study

All procedures were performed under ketamine and xylazine anesthesia, and all efforts were made to minimize suffering. Following recovery from anesthesia, animals were fed once per day using CMK-2 (CLEA Japan, Inc., Tokyo, Japan). Drinking water was made available *ad libitum*. Animals were singly housed under conditions of controlled humidity (40 ± 5%), temperature (25 ± 1 °C), and light (12-h light/12-h dark cycle, lights on at 8:00 AM). Three- to five-year-old female cynomolgus macaques (2.5–3.5 kg each) from the Philippines (Ina Research, Inc., Ina, Japan) were used in this study. The macaques were confirmed as free from hepatitis B virus, hepatitis E virus, *Mycobacterium tuberculosis, Shigella* spp., *Salmonella* spp., and *Entamoeba histolytica*. In the text and figures, individual macaques are distinguished by identification numbers. The absence of anti-influenza A virus antibodies in the sera of these animals was confirmed before experiments using ELISA against purified HA protein.

The macaques were inoculated intradermally (in both upper arms using bifurcated needles) with 1 × 10^7^ PFU (5 × 10^6^ PFU/arm × 2) of rVV-H5 HA or rVV-Empty. Macaques inoculated with rVV-Empty were used as controls. Sera were collected every week for use in HI assays and *in vitro* neutralization assays. At 4 weeks after vaccination, the macaques were inoculated by intratracheal, intranasal, and tonsillar routes with a total of 3 × 10^6^ PFU of H5N1 A/whooper swan/Hokkaido/1/08 or 3 × 10^6^ TCID_50_ of H1N1 A/Narita/1/2009. Vaccination and challenge experiments using macaques were performed in the biosafety level 3 facility of the Research Center for Animal Life Science, Shiga University of Medical Science. Blood for sera for measurement via HI assay and *in vitro* neutralization assay was collected every day. Clinical signs were monitored for 7 days post-challenge; macaques were sacrificed on day 7 post-challenge.

### *In vitro* virus neutralization assay

Sera were inactivated at 56 °C for 30 min. For macaque sera, samples were subjected to serial 4-fold dilutions, and aliquots of each resulting dilution were mixed with 50 PFU of H5N1 A/whooper swan/Hokkaido/1/2008 or H1N1 A/Narita/1/2009 viruses. For mouse sera, samples were subjected to serial 4-fold dilutions, and aliquots of each dilution were mixed with 50 PFU of H5N1 A/whooper swan/Hokkaido/1/2008 or H1N1 A/Tokyo/2619/2009 viruses. After incubation for 1 h at 37 °C, each mixture was used to inoculate one well of a 6-well plate seeded with MDCK cells; the plates then were incubated for an additional hour at 37 °C (H5N1 HPAI virus) or 34 °C (H1N1 pdm virus). After washing with MEM supplemented with 0.11% Na bicarbonate and 2 mM L-glutamine, the MDCK cells were overlaid (2 ml/well) with 0.8% agarose in MEM supplemented with 1% BSA, 2 mM L-glutamine, vitamins, and 10 μg/mL acetylated trypsin. Overlaid plates were placed in a biological safety cabinet until the overlay solidified, then shifted back to the 37 °C incubator (H5N1 HPAI virus) or 34 °C incubator (H1N1 pdm virus). After 3 days of incubation, the cells were fixed with 10% neutral buffered formalin. Viral titers were determined by counting plaque numbers. Neutralization titers were expressed as the reciprocal of the maximum dilution of serum at which plaque numbers were reduced two-fold (50%) compared to wells infected with virus in the absence of serum.

### Hemagglutination inhibition (HI) assay

HI assays were performed according to the standard method^41^. Briefly, serum samples were pretreated (37 °C, 18 h) with a receptor-destroying enzyme (RDE II, Denka Seiken, Tokyo, Japan), and then heat inactivated (56 °C, 30 min). The resulting serum samples were subjected to serial 2-fold dilutions with 5 mM Na phosphate buffer (pH 7.2) and then mixed with a 4 × hemagglutination (HA) titer of H5N1 A/whooper swan/Hokkaido/1/2008 or H1N1 A/Narita/1/2009 (or A/Tokyo/2619/2009) in a 0.5% suspension of chicken erythrocytes. After 1 h incubation at room temperature, hemagglutination was judged by visual inspection; titers were expressed as reciprocal of the maximum dilution of serum that completely inhibited hemagglutination.

### Lung histopathology and inflammation scores

The upper lobe of the right lung from each necropsied mouse was fixed in 10% neutral buffered formalin, embedded in paraffin, sectioned at 4-μm thicknesses, stained with hematoxylin and eosin (H-E), and subjected to routine histological examination. Histopathological findings were evaluated according to the modified methods of the previous study^42^. The grading system for the histological changes was as follows. For each lung tissue section, 10 randomly selected microscopic fields were scanned at a magnification of 100×, and each field was graded visually on a scale from 0 to 7, defined as follows: 0, normal lung; 1, mild destruction of epithelium in trachea and bronchus; 2, mild infiltration of inflammatory cells around the periphery of bronchiole; 3, moderate infiltration of inflammatory cells around the alveolar walls, resulting in alveolar thickening; 4, mild alveolar injury accompanied by vascular damage (<10%); 5, moderate alveolar and vascular injury (10–30%); 6, severe alveolar injury with hyaline membrane-accompanied alveolar hemorrhage (<50%); and 7, severe alveolar injury with hyaline membrane and alveolar hemorrhage (>50%). The mean value of the grades obtained for all fields from a given animal was used as the grade of visual lung injury in that animal.

### Immunohistochemical analyses

Spleens of mice and macaques were embedded in FSC 22 Frozen Section Compound (Leica Microsystems, Wetzlar, Germany) and frozen immediately in hexane chilled with dry ice. The frozen block was sliced into 6-μm-thick sections using a Cryostat CM1950 (Leica Microsystems). Sections were fixed with a mixture of acetone/methanol, blocked with BlockAce (DS Pharma Biomedical, Osaka, Japan), and then stained with biotin-conjugated (bio)-peanut agglutinin (PNA) (Vector Laboratories, Burlingame, CA), anti-IgD monoclonal antibody (mAb), alkaline phosphatase-conjugated anti-rat IgG (SouthernBiotech, Birmingham, AL), and horseradish peroxidase (HRP)-conjugated streptavidin (KPL, Gaithersburg, MD). Signals were developed using a Vector Blue AP Substrate Kit (Vector Laboratories) and 3,3′-diaminobenzidine tetrahydrochloride (DAB) substrate (DOJINDO, Kumamoto, Japan), and staining was observed using a BZ-X700 microscope (Keyence, Osaka, Japan). The number of mature GCs surrounded by B-cell follicles was counted on each slide. For immunofluorescence staining, sections were blocked with TNB Blocking buffer and stained with the following reagents: allophycocyanin (APC) -anti-CD11c mAb, biotin-anti-CD3ε mAb (BioLegend, San Diego, CA), Alexa488-anti-B220 mAb, APC-anti-CD11b mAb (eBioscience, San Diego, CA), purified anti-Follicular Dendritic Cell mAb (BD Biosciences), Alexa594-anti-Rat IgG mAb (Life Technologies), and Alexa 488-conjugated streptavidin (Invitrogen, Carlsbad, CA). The signal was observed using a BZ-X700 microscope or an Airyscan-enabled LSM880 confocal microscope (Carl Zeiss, Jena, Germany). The area of CD11c^+^ -staining spots was calculated using a BZ-X Analyzer at a magnification of 200×. To allow visualization of the interaction between dendritic cells and T-cells and to enhance the image clarity, the confocal data acquired by the Airyscan-enabled LSM880 at a magnification of 630× was reconstituted by Imaris software (Carl Zeiss). The spleen from each mouse challenged with H5N1 HPAI virus following vaccination with rVV-H5 HA or rVV-empty was fixed in 10% neutral buffered formalin, embedded in paraffin, tissue sectioned at 6-μm thicknesses, and then stained with APC-anti-CD11c mAb, biotin-anti-CD3ε mAb, and Alexa 488-conjugated streptavidin.

### Cell preparation

Single-cell suspensions were prepared from the spleens and the lungs as described previously^43^. Briefly, spleen samples were prepared by passing the tissues through a nylon screen (95 μm, Flon Industry). For isolation of lung cells, mice were perfused with phosphate-buffered saline (PBS) in the right ventricle to clear the blood. Lungs were minced and incubated at 37 °C for 80 min in 2 mg/ml collagenase D (Roche) and 10 μg/ml DNase I (Roche), and then disrupted between the frosted ends of glass slides. After centrifugation on a 70%/44%/30% Percoll gradient, the cells at the 70%/44% interface were recovered.

### Flow cytometry

Mouse splenocytes were stained with antibodies to B220, GL-7, CD11c, CD11b, F4/80 and ICAM-1 (all BioLegend), Fas (BD biosciences), and FDC-M2 (ImmunoKontact). Fixable Viability Stain 510 (BD biosciences) was used for all flow cytometry experiments. Mouse lung cells were stained with antibodies to CD11c, CD11b and F4/80 (all BioLegend). Splenocytes and lung cells were fixed using the Transcription Factor Buffer Set (BD biosciences) according to the manufacturer’s protocol. All samples were run on an LSR Fortessa (BD biosciences) and analyzed using FlowJo (Tree Star).

### Statistical analyses

Data are presented as mean ± standard deviation (SD), where applicable. Inferential statistical analysis was performed by Student’s t-test, Welch’s t-test, and One-Way ANOVA followed by Tukey’s test. *p* values < 0.05 were considered statistically significant.

## Additional Information

**How to cite this article**: Yasui, F. *et al*. Sensitization with vaccinia virus encoding H5N1 hemagglutinin restores immune potential against H5N1 influenza virus. *Sci. Rep.* **6**, 37915; doi: 10.1038/srep37915 (2016).

**Publisher's note:** Springer Nature remains neutral with regard to jurisdictional claims in published maps and institutional affiliations.

## Supplementary Material

Supplementary Information

Supplementary Movie 1

Supplementary Movie 2
