# Supplementary material for: Sensitization with vaccinia virus encoding H5N1 hemagglutinin restores immune potential against H5N1 influenza virus
Source: Sci Rep. 2016 Nov 28;6:37915. doi: 10.1038/srep37915 (PMC5124960; doi:10.1038/srep37915)
Supplement: Supplementary Information [file srep37915-s1.pdf]

SUPPLEMENTARY INFORMATION

Sensitization with vaccinia virus encoding H5N1 hemagglutinin restores immune potential against H5N1 influenza virus

Fumihiko Yasui[1†], Yasushi Itoh[2†], Ai Ikejiri[1†], Masahiro Kitabatake[3], Nobuo Sakaguchi[1,5], Keisuke Munekata[1], Shintaro Shichinohe[2], Yukiko Hayashi[1], Hirohito Ishigaki[2], Misako Nakayama[2], Yoshihiro Sakoda[4], Hiroshi Kida[4], Kazumasa Ogasawara[2], Michinori Kohara[1*]

† These authors contributed equally to this work.

Corresponding author: Dr. Michinori Kohara

Tel: +81-3-5316-3232; Fax: +81-3-5316-3137;

E-mail address: kohara-mc@igakuken.or.jp

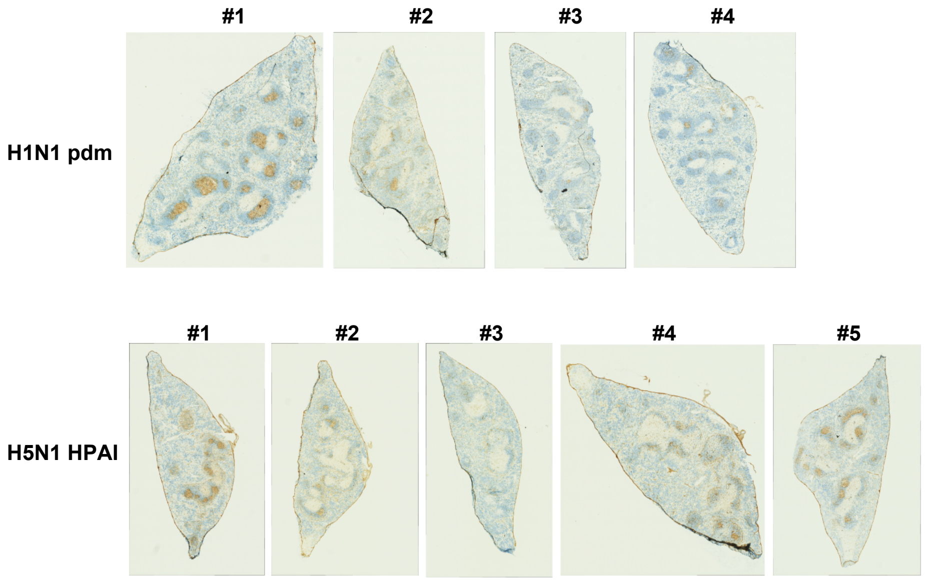

Supplementary figure 1. Immunohistological staining of germinal centers (GCs) and B-cell follicles in the spleens of BALB/c mice infected with either H5N1 HPAI virus or H1N1 pdm virus.

The GCs and B-cell follicles in the spleens of the mice infected with either H5N1 HPAI virus or H1N1 pdm virus were imaged following stain with PNA (brown; GCs) and anti-IgD antibody (blue; B-cells). Representative images are shown; numbers (#s) indicate individual animal numbers.

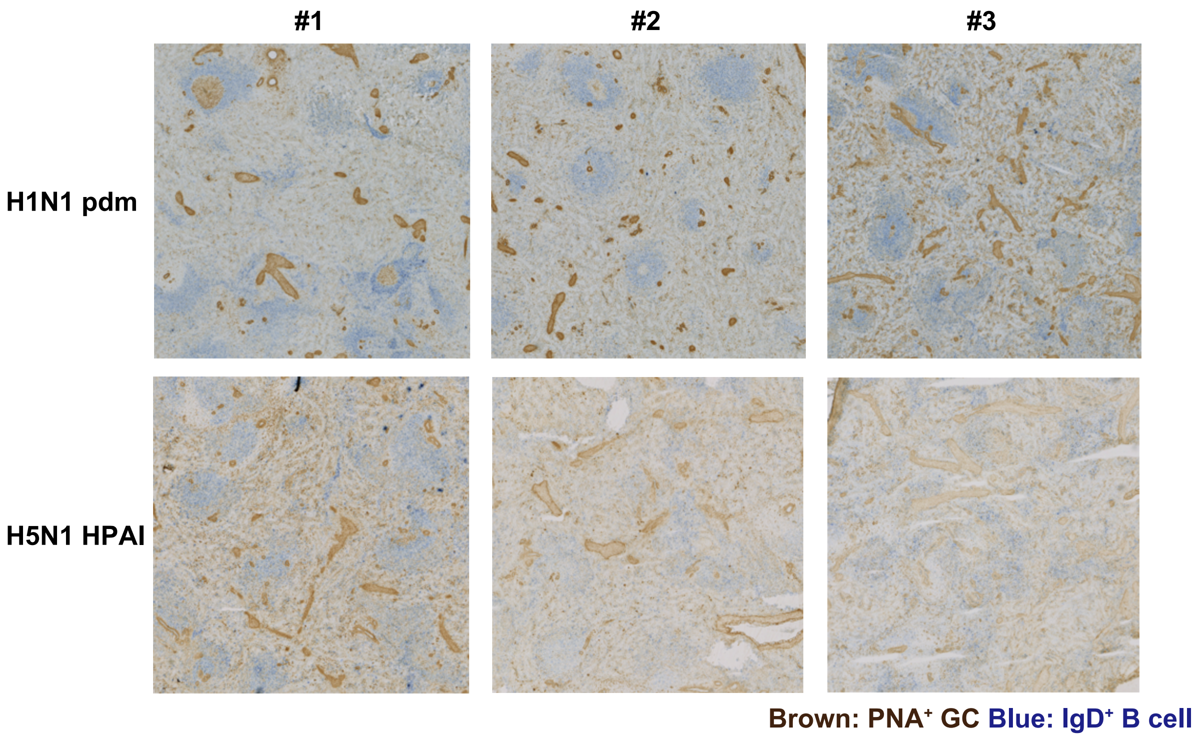

Supplementary figure 2. Immunohistological staining of germinal centers (GCs) and B-cell follicles in the spleens of macaques infected with either H5N1 HPAI virus or H1N1 pdm virus.

The GCs and B-cell follicles in the spleens of the macaques infected with either H5N1 HPAI virus or H1N1 pdm virus were imaged following staining with PNA (brown; GCs) and anti-IgD antibody (blue; B-cells). Representative images are shown; numbers (#s) indicate individual animal numbers.

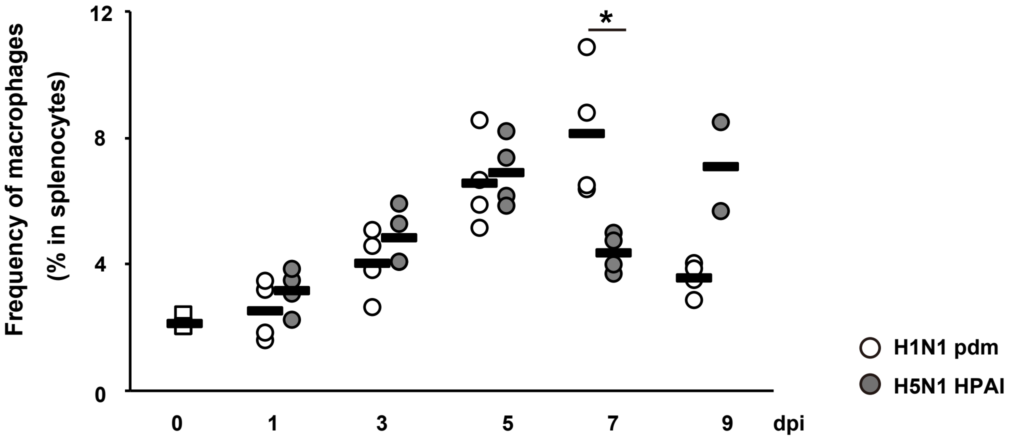

Supplementary figure 3. FACS analysis of macrophages from the spleen of mice infected with either H5N1 HPAI virus or H1N1 pdm virus.

The percentage of macrophages ($CD11c^{low}$, $CD11b^{high}$, $F4/80^{+}$) in the spleens of mice infected with either H5N1 HPAI virus or H1N1 pdm virus is shown following analysis by flow cytometry at 0, 1, 3, 5, 7 and 9 dpi (n = 4 for all except n = 2 in H5N1 HPAI virus-infected mice at 9 dpi). * ; $p < 0.05$

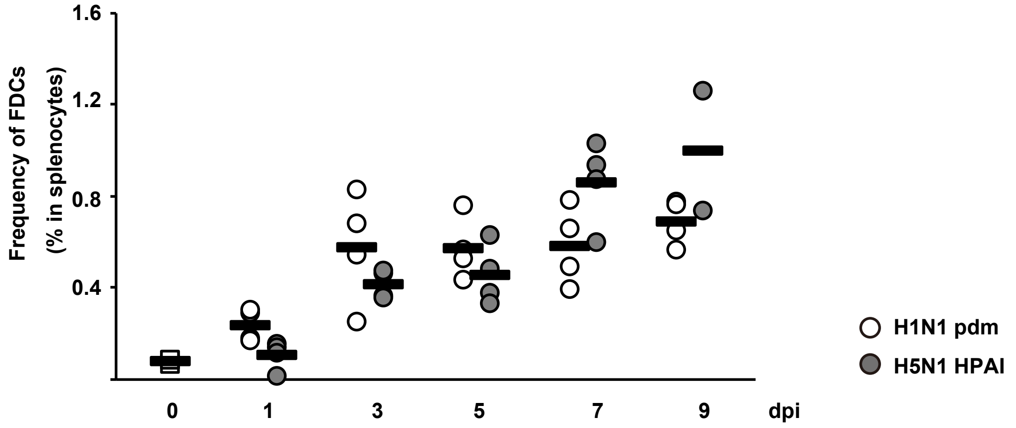

Supplementary figure 4. FACS analysis of follicular dendritic cells (FDCs) in the spleens of mice infected with either H5N1 HPAI virus or H1N1 pdm virus.

The percentage of FDCs (FDC-M2$^+$, ICAM-1$^+$) in the spleens of mice infected with either H5N1 HPAI virus or H1N1 pdm virus is shown following analysis by flowcytometry at 0, 1, 3, 5, 7 and 9 dpi (n = 4 for all except n = 2 in H5N1 HPAI virus-infected mice at 9 dpi).

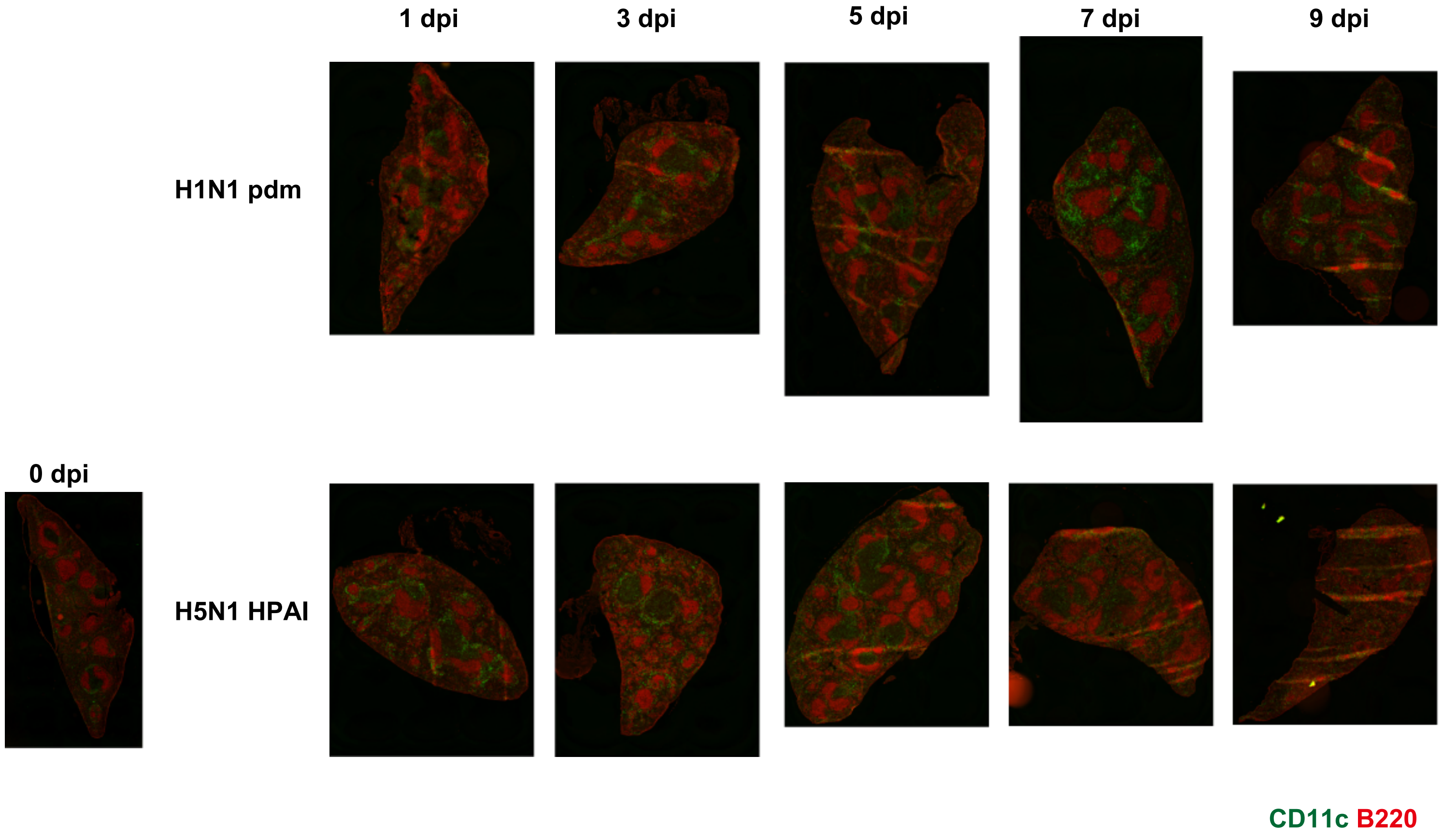

Supplementary figure 5. Immunofluorescence staining of dendritic cells and B-cells in the murine spleens recovered at 0, 1, 3, 5, 7, or 9 dpi with either H5N1 HPAI virus or H1N1 pdm virus.

The accumulation of dendritic cells around B-cell follicles in the spleens of mice infected with either H5N1 HPAI virus or H1N1 pdm virus was visualized by staining with CD11c (green) and B220 (red). Images are representative data collected from two or three independent experiments (n = 3-5 per time point).

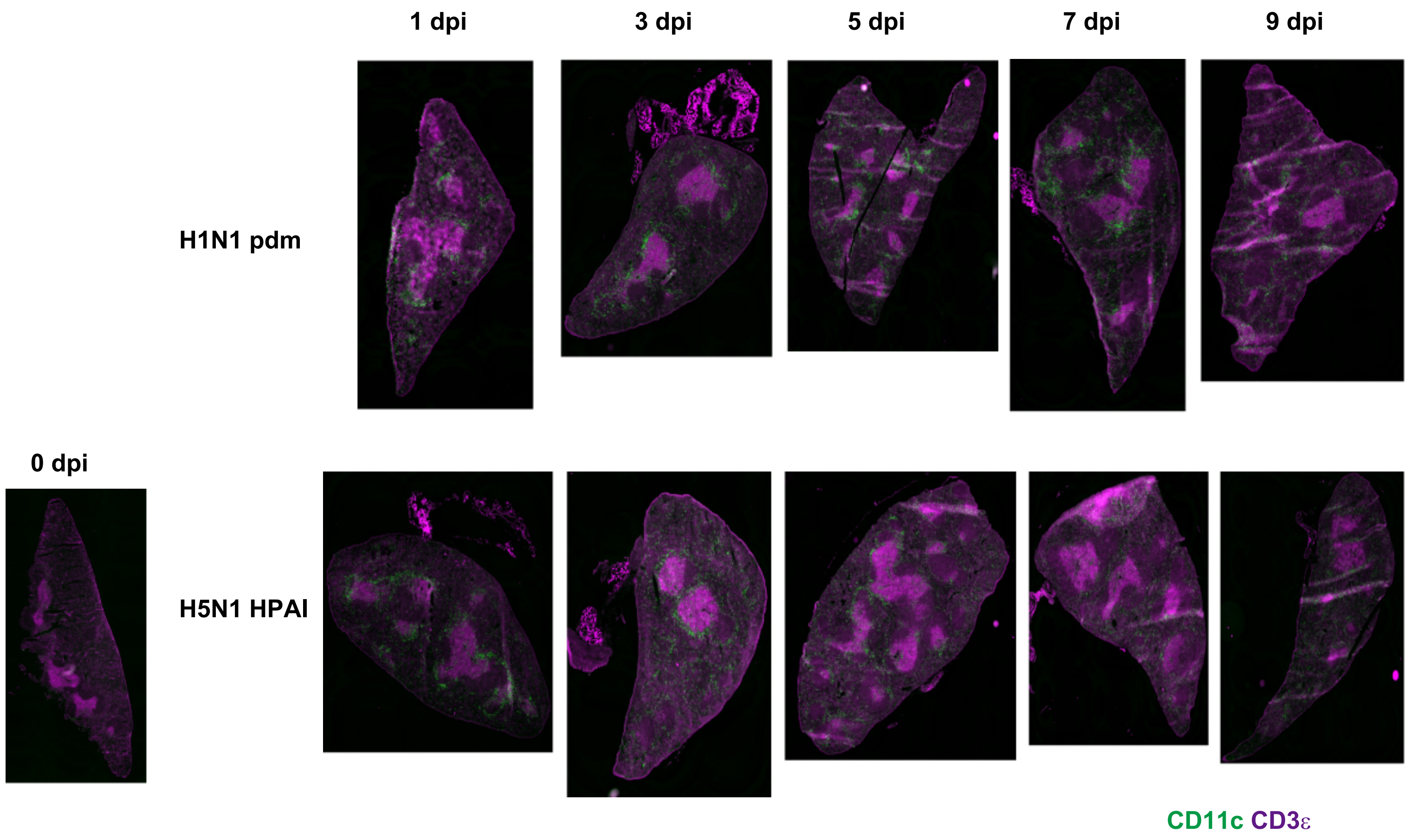

Supplementary figure 6. Immunofluorescence staining of dendritic cells and T-cells in the murine spleens recovered at 0, 1, 3, 5, 7, or 9 dpi with either H5N1 HPAI virus or H1N1 pdm virus.

The accumulation of dendritic cells in the T-cell zone in the spleens of mice infected with either H5N1 HPAI virus or H1N1 pdm virus is shown by staining with CD11c (green) and CD3ε (magenta), respectively. Images are representative data collected from two or three independent experiments (n = 3-5 per time point).

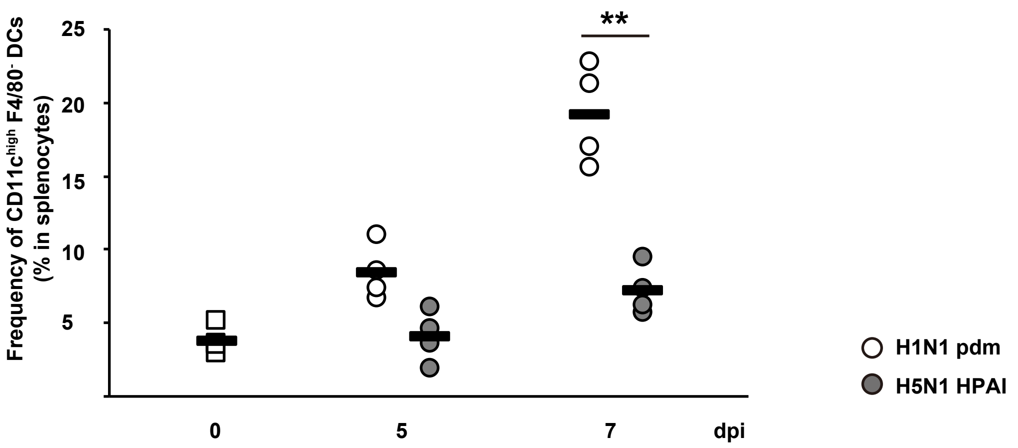

Supplementary figure 7. FACS analysis of CD11c^{high} F4/80^{-} dendritic cells in the lungs of mice infected with either H5N1 HPAI virus or H1N1 pdm virus.

The percentage of dendritic cells (CD11c^{high}, F4/80^{-}) in the lungs of mice infected with either H5N1 HPAI virus or H1N1 pdm virus is shown following analysis by flow cytometry (n = 4). ** ; $p < 0.01$

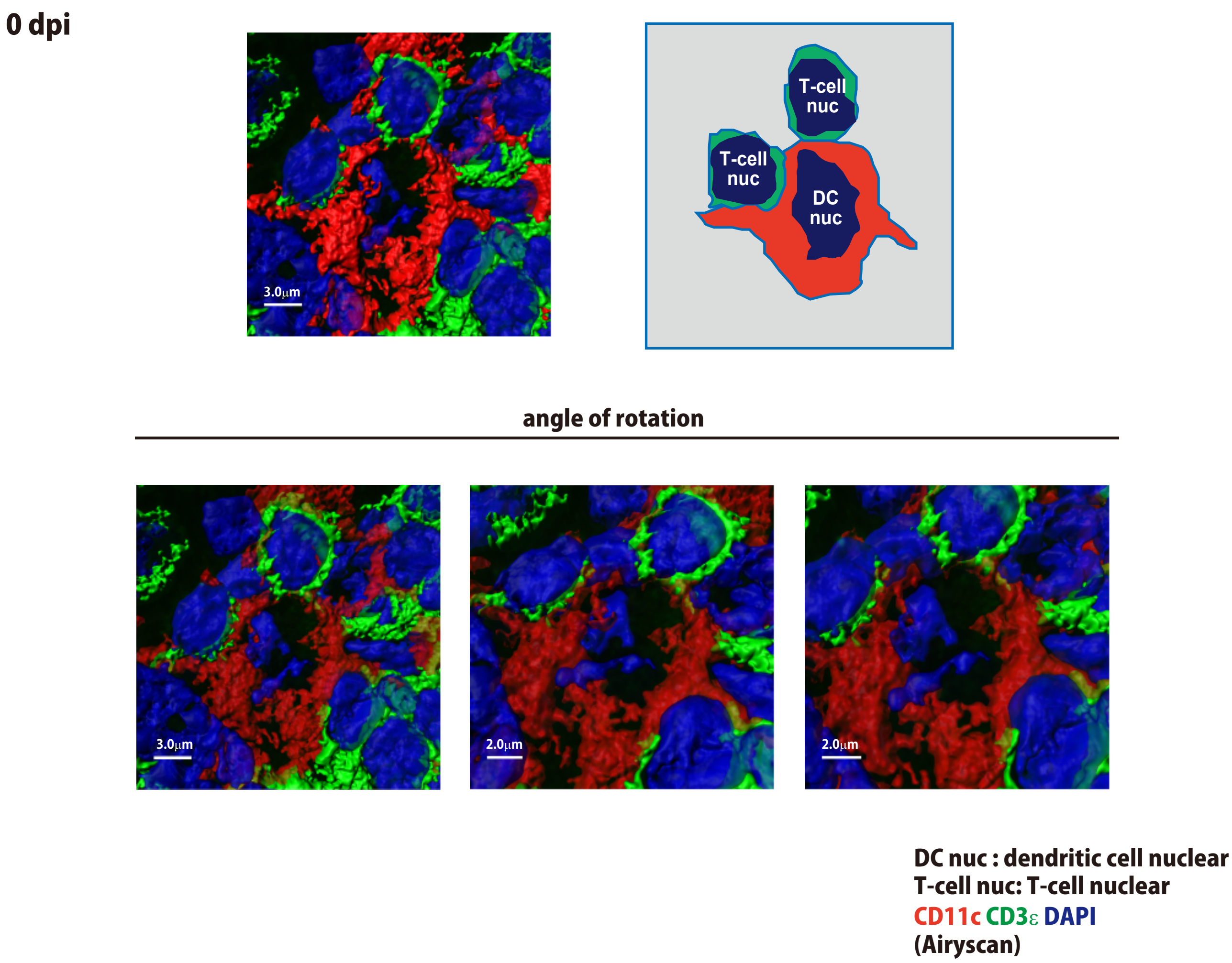

Supplementary figure 8. Cell-cell interaction between dendritic cells and T-cells in naive murine spleens observed by high-resolution microscopy.

High-resolution micrographs were obtained from sections of the naive murine spleen. Images were visualized by Imaris software and are representative data. Sections were stained for CD11c (red), CD3ε (green) and nuclear DNA (DAPI; blue). To clarify the the interaction of dendrtic cells with T-cells, the rotation angle was changed (lower pannels). To enhance image clarity, the transparency of the CD11c rendering was set to 60% (lower pannels) and the transparency of DAPI was set to 78% (all pannels).

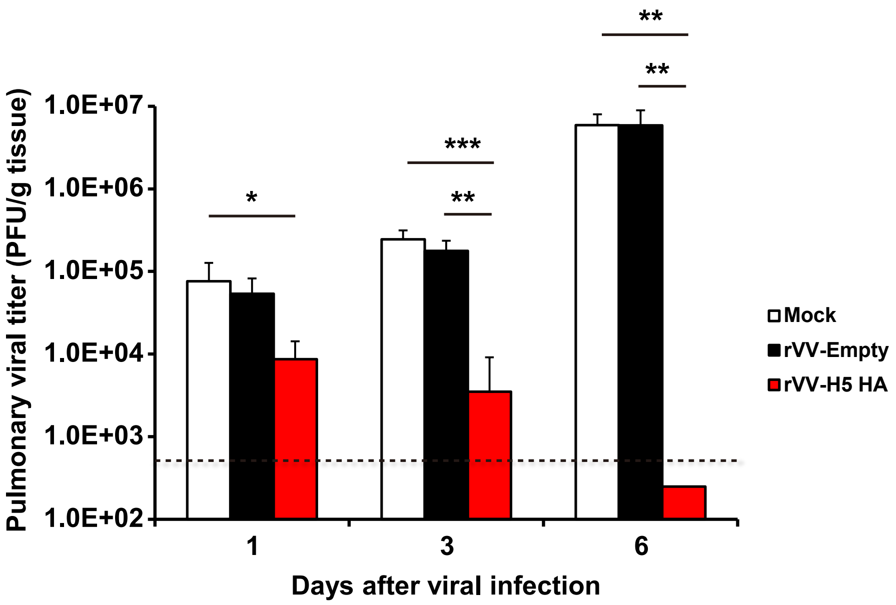

Supplementary figure 9. Viral titers in the lungs after H5N1 HPAI virus infection

Nine-week-old female BALB/c mice were inoculated intradermally with either rVV-Empty (black column) or rVV-H5 HA (red column) at 1×10^{7} PFU per animal.

Phosphate-buffered saline (PBS)-treated mice were used as the mock group (white column). At 5 weeks after vaccination, the mice were infected intranasally with H5N1 HPAI virus (A/whooper swan/Hokkaido/1/2008) at 1×10^{4} PFU per animal. The viral tier in the lungs was measured at the indicated time points. Broken lines indicate the detection limit in the assay. *p* values were calculated via One-Way ANOVA and post-hoc Tukey' s test. * $p < 0.05$, ** $p < 0.01$, and *** $p < 0.001$.

Supplementary movie 1. Three-dimensional image of the co-immunohistological staining of dendritic cells and T-cells in the murine spleen at 7 days after H1N1 pdm virus infection.

Representative high-resolution micrographs of the spleen of the mice infected with H1N1 pdm virus at 7 dpi were acquired using an Airyscan-enabled LSM880 confocal microscope and visualized as a three-dimensional image by Imaris software. Staining was performed for CD11c (red), CD3ε (green), and nuclear DNA (DAPI; blue). To clarify the interaction of dendritic cells with T-cells, the angle of rotation is changed over the course of the movie. To enhance image clarity, the transparency of CD11c rendering was set to 60% and the transparency of DAPI was set to 78%.

Supplementary movie 2. Three-dimensional image of the co-immunohistological staining of dendritic cells and T-cells in the murine spleen at 7 days after H5N1 HPAI virus infection.

Representative high-resolution micrographs of the spleen of the mice infected with H5N1 HPAI virus at 7 dpi were acquired using an Airyscan-enabled LSM880 confocal microscope and visualized as a three-dimensional image by Imaris software. Staining was performed for CD11c (red), CD3ε (green), and nuclear DNA (DAPI; blue). To clarify the interaction of dendritic cells with T-cells, the angle of rotation is changed over the course of the movie. To enhance image clarity, the transparency of CD11c rendering was set to 60% and the transparency of DAPI was set to 78%.
